# Assessment of anxiety and psychological stress among expectant parents during pregnancy: a scoping review

**DOI:** 10.1590/0034-7167-2024-0587

**Published:** 2025-12-08

**Authors:** Inês Santos Tinta, Maria Helena de Carvalho Valente Presado, Sandra Margarida Gaiato Risso

**Affiliations:** I Centro de Investigação, Inovação e Desenvolvimento de Enfermagem de Lisboa. Lisboa, Portugal; II Escola Superior de Enfermagem de Lisboa. Lisboa, Portugal

**Keywords:** Anxiety, Risk Assessment, Pregnancy, Patient Health Questionnaire, Psychological Stress., Ansiedad, Evaluación del Riesgo, Embarazo, Cuestionario de Evaluación del Paciente, Estrés Psicológico.

## Abstract

**Objectives:**

to map, in the scientific literature, instruments used to assess anxiety and stress among expectant parents during pregnancy.

**Methods:**

we conducted a scoping review guided by the JBI and PRISMA-ScR guidelines, searching the databases CINAHL, MEDLINE, Cochrane, Scopus, ScienceDirect, Web of Science, and the Open Access Scientific Repositories of Portugal. Quantitative studies were included with no restrictions on time or language.

**Results:**

we identified 16 instruments (10 for anxiety and 6 for stress), predominantly applied to pregnant women in the third trimester.

**Conclusions:**

studies involving expectant fathers are scarce. To better understand their feelings and needs, it is essential to apply instruments that assess anxiety and stress, thereby supporting anticipatory care. Developing and validating instruments with expectant parents (both men and women) across the trimesters of pregnancy will broaden professionals’ and the public’s knowledge on this issue.

## RESUMEN

**Objetivos:**

mapear, en la literatura científica, instrumentos para evaluar la ansiedad y el estrés de los futuros padres durante el embarazo.

**Métodos:**

revisión de alcance realizada siguiendo la metodología del JBI y las directrices del PRISMA-ScR, en las bases de datos CINAHL, MEDLINE, COCHRANE, Scopus, *ScienceDirect, Web of Science* y Repositorios Científicos de Acceso Abierto de Portugal. Se incluyeron estudios cuantitativos sin restricción temporal ni idiomática.

**Resultados:**

se identificaron 16 instrumentos (10 para evaluar la ansiedad y 6 para el estrés), aplicados principalmente a mujeres embarazadas en el tercer trimestre.

**Conclusiones:**

los estudios con futuros padres varones son escasos. Para comprender mejor sus sentimientos y necesidades, es fundamental aplicar instrumentos de evaluación de la ansiedad y el estrés, posibilitando una atención anticipatoria. Desarrollar y/o validar instrumentos con futuros padres (hombres y mujeres) en los distintos trimestres del embarazo permitirá ampliar el conocimiento de los profesionales sanitarios y de la población.

## INTRODUCTION

Pregnancy is often perceived as a joyful period; however, scientific literature shows that expectant parents are increasingly experiencing higher levels of anxiety and stress, which may negatively affect their physical and mental health as well as neonatal outcomes^(1)^. Pregnancy involves the transition to parenthood, which can generate many concerns for couples and have important impacts on their health and well-being. Moreover, the literature has primarily focused on the functional aspects of parenthood, while neglecting those related to parents’ psychological well being^(2)^. Their well-being is influenced by how they feel and how they experience certain emotions, particularly anxiety and stress.

Anxiety is often mistakenly used interchangeably with stress; however, this is not correct, as anxiety is defined as an emotional response to anticipated danger, whose origin is unknown or unrecognized^(3)^. The degree of anxiety experienced is related to certain predisposing factors, one of which is the individual’s current health status.

Stress, on the other hand, is a state of imbalance between the demands placed on an individual, whether internal or external, and the individual’s capacity to cope with these demands^(3)^. It is triggered by both negative and positive experiences that require adjustments in response to changes in daily life and may even contribute to the etiology of certain diseases.

Stress can therefore be considered an external stressor, while anxiety is the subjective emotional response to that stressor.

Pregnancy itself can be understood as a stressor, as it is characterized by neuroendocrine, cardiovascular, and immunological changes^(4)^. Expectant parents are at greater risk of developing emotional and mental health problems during pregnancy and in the first year after childbirth^(5)^.

Screening for anxiety and stress is essential, since maternal and paternal anxiety during pregnancy increases the likelihood of behavioral problems in children, with maternal anxiety having the greatest influence on outcomes^(6)^. However, paternal stress during this period also directly affects child development^(7)^. Among expectant mothers, approximately 40% present symptoms of anxiety and identify the possibility of stress disorders during pregnancy^(4,8)^. The father’s role also directly affects the stress levels of pregnant women, showing the mutual influence of feelings^(9)^.

Expectant fathers often consider pregnancy a difficult period, marked by emotions such as fear and anxiety, which may negatively affect their relationship not only with their partners but also later with their children^(10)^. Therefore, paternal stress should also be considered and assessed during pregnancy, as this represents a critical window for early intervention to prevent complications^(11)^.

Researchers recommend implementing actions that include screening for anxiety and stress in fathers to provide appropriate preventive and therapeutic interventions. This recommendation is supported by evidence showing that about 25% experience symptoms of anxiety during their partners’ pregnancy, with the origins of these feelings spanning several domains: concerns about childbirth, the transition to fatherhood, and the baby; relationship with the partner; attitudes toward healthcare professionals involved in the process; and financial concerns^(11,12)^.

Fathers can help protect against the development of maternal mental health problems resulting from stress and anxiety during pregnancy, provided their own mental health is supported through the promotion of well-being^(13)^. As previously mentioned, the presence of these feelings in expectant parents during pregnancy influences child behavior; therefore, addressing the couple’s mental health through early identification benefits the family as a whole. In addition, men often report being reluctant to express their feelings or their need for help because they prioritize their partners’ needs and feel excluded from health services^(13)^. This sense of exclusion stems from a lack of human and healthcare resources tailored to men in the broader context of fatherhood and mental health, which are essential for promoting their self-care and their role as involved caregivers^(13)^.

This gap in nursing practice underscores the need to identify instruments that can be applied in a timely manner to assess and detect anxiety and stress in parents during pregnancy, thereby enabling care practices that foster a positive experience of this important period in couples’ lives. To address this issue, the most appropriate research method is the scoping review, as it allows mapping and synthesizing the existing scientific evidence as well as identifying knowledge gaps^(14)^.

It should be noted that a preliminary search in the JBI and in the databases MEDLINE Ultimate^®^ (EBSCOhost) and the Cochrane Database of Systematic Reviews^®^ (EBSCOhost) did not identify any current or ongoing systematic or scoping reviews on this topic.

## OBJECTIVES

To map, in the scientific literature, instruments used to assess anxiety and stress among expectant parents during pregnancy.

## METHODS

### Study design and period

This scoping review was conducted according to the JBI methodology for scoping reviews and reported following the Preferred Reporting Items for Systematic Reviews and Meta-Analyses extension for Scoping Reviews (PRISMA-ScR)^(14)^. A protocol was developed to define the objectives, methods (including sources of evidence, exclusion criteria, data extraction and presentation methods, and evidence analysis), and the search strategy. The protocol was registered in the Open Science Framework Registries (OSF) on April 26, 2024, and is available at https://doi.org/10.17605/OSF.IO/EB3FA. It underwent some changes, particularly regarding the exclusion criteria, with two additional criteria included that were not mentioned in the initial protocol (COVID-19 pandemic and addictive behaviors). The search for this scoping review was conducted on April 30, 2024, and updated on April 15, 2025, with no additional studies identified that met the inclusion criteria and addressed the research question.

The search strategy aimed to identify published and unpublished studies, with no time or language restrictions. An initial limited search of MEDLINE Ultimate^®^ and CINAHL Ultimate^®^ was conducted to identify relevant articles. Words contained in the titles and abstracts of these articles, as well as keywords and indexed terms, were then used to develop a comprehensive search strategy across the Cochrane Database of Systematic Reviews, ScienceDirect^®^, Scopus^®^, and Web of Science^®^. Sources of unpublished studies/gray literature included the Open Access Scientific Repositories of Portugal, electronic scientific articles, and digital books. The inclusion of gray literature reduces susceptibility to bias by considering all available data on the subject^(15)^. The following search strategy was applied: (“Future Parents” OR “MH Expectant Mothers” OR “MH Expectant Fathers” OR “MH Expectant Parents”) AND ((“Anxiety” OR “MH Anxiety”) OR (“Estresse” OR “MH Phychological Estresse” OR “MH Estresse”)) AND (“Pregnancy” OR “Pregnan*” OR “MH Pregnancy”) AND (“Assessment instrument” OR “Assessment tool” OR “Scale*” OR “Questionnaire” OR “Form” OR “MH Risk Assessment”). The search expression included truncations, namely pregnan* and scale*, to capture as many articles on the subject as possible.

Regarding the inclusion criteria, the Participants, Concept, and Context (PCC) strategy was adopted: participants were expectant parents; the concepts were anxiety, stress, and pregnancy; and the context considered was studies that included assessment instruments.

All scientific articles on the topic were eligible, specifically quantitative studies, published or unpublished, with experimental and quasi-experimental designs. These included randomized and non-randomized controlled clinical trials, before-and-after studies, interrupted time series studies, case-control studies, analytical or descriptive cross-sectional studies, development and validation studies, and systematic reviews that met the inclusion criteria. Opinion papers were also considered, as well as articles in any language and with no time restrictions.

Mixed-methods studies, letters to the editor, conference abstracts, incomplete articles, and ongoing studies were excluded. Studies were also excluded if the concept addressed depression, postpartum depression, pathological stress, or pathological anxiety; pregnancies with induced or associated pathology; pregnancies resulting from medically assisted reproduction techniques; or fetuses with pathology diagnosed in utero. In addition, studies addressing the COVID-19 pandemic or addictive behaviors were excluded.

In the third stage, after the search, all identified citations were gathered and uploaded to Rayyan^®^, and duplicates were removed. Two independent reviewers screened the titles and abstracts against the inclusion criteria. Potentially relevant sources were retrieved in full. Again, two independent reviewers thoroughly assessed the full texts of the selected citations according to the inclusion criteria. Discrepancies were resolved without the need for a third reviewer.

## RESULTS

**Figure 1:**
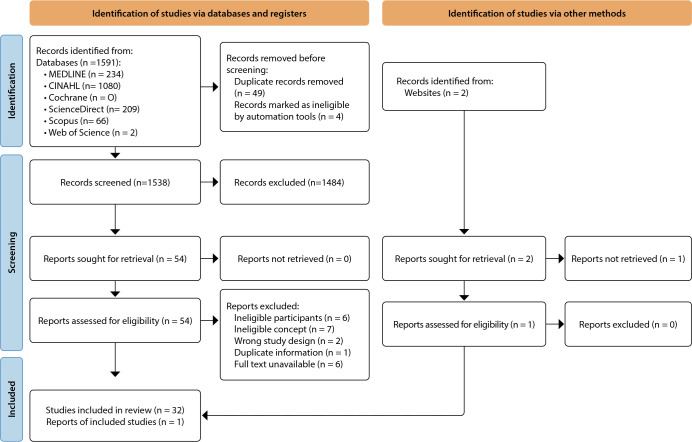
Flow diagram based on the Preferred Reporting Items for Systematic Reviews and Meta-Analyses Extension for Scoping Review

A total of 1,591 studies were identified. After transfer to the Rayyan^®^ application, 49 duplicates were removed, and 4 studies were automatically excluded based on ineligibility criteria, resulting in 1,538 studies for screening. Two additional studies were identified in the gray literature; however, one was excluded in the first screening phase. After applying the inclusion and exclusion criteria, 1,484 studies were excluded, and 55 were retained for full-text review, of which 22 were excluded. Therefore, this scoping review includes 33 studies. The search results and the process of inclusion and exclusion are presented in Figure 1 as a flow diagram based on the Preferred Reporting Items for Systematic Reviews and Meta-Analyses extension for Scoping Reviews (PRISMA-ScR).

The data extracted from the included studies were organized in a data extraction table developed and adapted according to JBI guidelines. This table includes the following information: author/year/country, objectives, participants, methods (study design), instruments for assessing anxiety, instruments for assessing stress, and psychometric properties.

The data extracted from the studies were organized according to the anxiety and stress assessment instrument identified and the trimester of pregnancy in which it was applied (first, second, or third trimester). The results are presented in figures, graphs, and tables.

### Date and place of publication

The included studies (n = 33) were published between 2004 and 2024. The number of publications increased from 2019 (n = 5) and peaked in 2022 (n = 7), highlighting the growth of scientific production on the topic and the continuous updating of the evidence.

**Figure 2:**
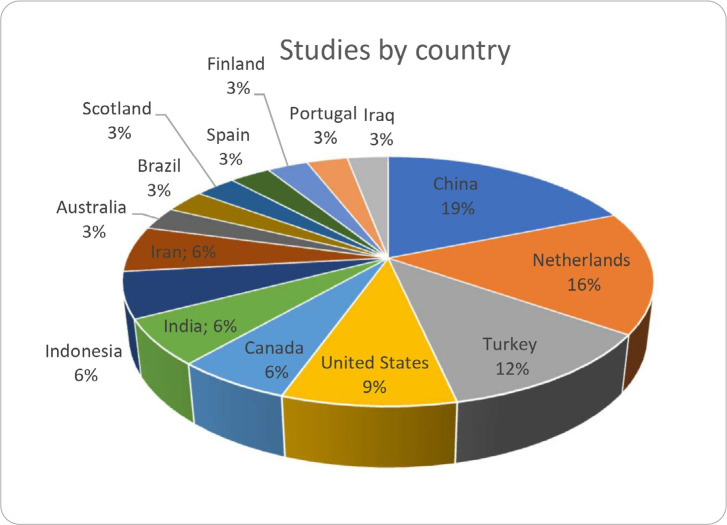
Distribution of the percentage of studies by country

The studies that included instruments for assessing anxiety and stress were conducted in several countries (Figure 2), namely China (n = 6; 19%), the Netherlands (n = 5; 16%), Turkey (n = 4; 12%), the United States (n = 3; 9%), Canada (n = 2; 6%), India (n = 2; 6%), Indonesia (n = 2; 6%), Iran (n = 2; 6%), Australia (n = 1; 3%), Brazil (n = 1; 3%), Scotland (n = 1; 3%), Spain (n = 1; 3%), Finland (n = 1; 3%), Portugal (n = 1; 3%), and Iraq (n = 1; 3%). Asia (represented by China, Turkey, India, and Indonesia) stood out as the continent with the greatest research interest in anxiety and stress.

### Types of studies

The studies analyzed used a quantitative approach. Most were longitudinal (n = 8), followed by correlational (n = 7), cross-sectional (n = 6), quasi-experimental (n = 3), experimental (n = 2), validation (n = 4), exploratory (n = 1), pilot (n = 1), and replication (n = 1) studies. Thus, the most common study designs were prospective longitudinal.

### Objectives of studies

The primary objective of the studies was to determine the prevalence of anxiety and stress in pregnant women across the three trimesters of pregnancy. Some studies compared, among pregnant women, levels of general and pregnancy-specific anxiety during pregnancy and in the first month of the child’s life with quality of life, life satisfaction, and potential associated factors such as cultural aspects, relationship with the partner, and sleep quality. They also examined predictors of stress, relating paternal stress to counseling during pregnancy and associating anxiety with information seeking about pregnancy. Some studies developed, translated, validated, and/or adapted instruments for the population of the country of origin, particularly in the Netherlands, Australia, Turkey, India, Canada, China, and the United States.

### Participants, concept, and context

Regarding participants, 31 studies included pregnant women, while only 2 included expectant fathers. Sample sizes ranged from 19 to 4,541, with one study including 10,002 participants.

**Figure 3:**
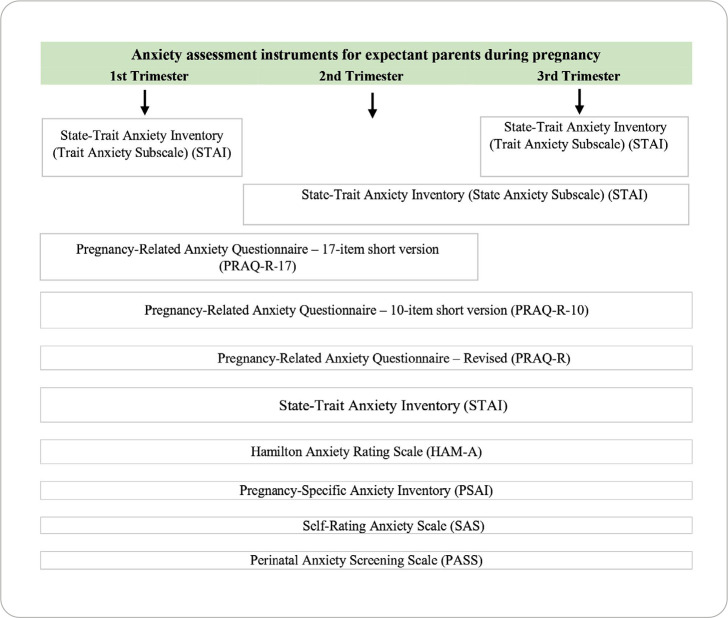
Distribution of the application of anxiety assessment instruments for expectant parents in the first, second, and third trimesters of pregnancy

**Figure 4:**
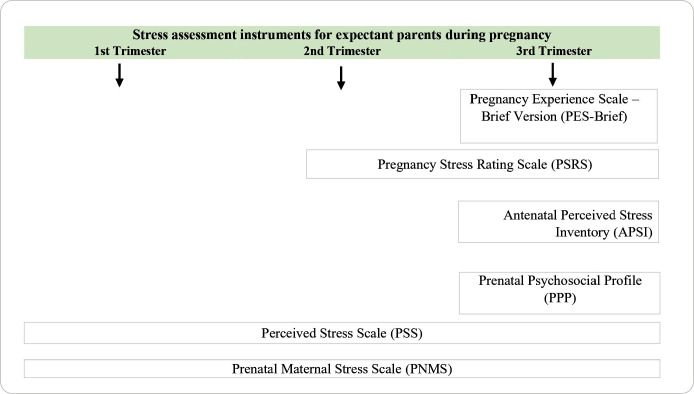
Distribution of the application of stress assessment instruments for expectant parents in the first, second, and third trimesters of pregnancy

Across the 33 studies included, researchers applied 16 instruments (described in detail below) to assess anxiety and stress in expectant parents during the three trimesters of pregnancy. Figure 3 presents the distribution of anxiety assessment instruments applied to expectant parents in the first, second, and third trimesters of pregnancy, while Table 1 provides a summary comparing these instruments in terms of applicability, internal consistency (Cronbach’s alpha), and trimester of application. Figure 4 presents the distribution of stress assessment instruments applied to expectant parents in the first, second, and third trimesters of pregnancy, while Table 2 summarizes these instruments in terms of applicability, internal consistency (Cronbach’s alpha), and trimester of application.

**Table 1:** Summary table comparing anxiety assessment instruments in terms of applicability, internal consistency (Cronbach’s alpha), and trimester of application

Instrument	Applicability		Internal consistency (Cronbach’s alpha)	Trimester of application		
	Women	Men		1^st^	2^nd^	3^rd^
**State-Trait Anxiety Inventory (STAI)**	x	x	0.83-0.94	x	x	x
**Trait Anxiety Subscale (STAI)**	x	x	Not reported	x		x
**State Anxiety Subscale (STAI)**	x		0.95		x	x
**Pregnancy-Related Anxiety Questionnaire - Revised (PRAQ-R)**	x		0.89-0.90	x	x	x
**PRAQ - 17-item short version**	x		0.69-0.76	x	x	
**PRAQ - 10-item short version (PRAQ-R-10)**	x		0.83-0.95	x	x	x
**Hamilton Anxiety Rating Scale (HAM-A)**	x		0.85	x	x	x
**Pregnancy-Specific Anxiety Inventory (PSAI)**	x		0.76	x	x	x
**Self-Rating Anxiety Scale (SAS)**	x		0.74-0.83	x	x	x
**Perinatal Anxiety Screening Scale (PASS)**	x		0.84-0.89	x	x	x

**Table 2:** Summary table comparing stress assessment instruments in terms of applicability, internal consistency (Cronbach’s alpha), and trimester of application

Instrument	Applicability		Internal consistency (Cronbach’s alpha)	Trimester of application		
	Women	Men		1^st^	2^nd^	3^rd^
**Pregnancy Experience Scale - Brief Version (PES-Brief)**	x		Not reported			x
**Pregnancy Stress Rating Scale (PSRS)**	x		0.92		x	x
**Antenatal Perceived Stress Inventory (APSI)**	x		0.70			x
**Prenatal Psychosocial Profile (PPP)**	x		Not reported			x
**Perceived Stress Scale (PSS)**	x		0.88-0.95	x	x	x
**Prenatal Maternal Stress Scale (PNMS)**	x		0.80	x	x	x

### State-Trait Anxiety Inventory (STAI)

The State-Trait Anxiety Inventory (STAI) was applied to pregnant women in the first^(16-20)^, second^(17-21)^, and third trimesters^(17-20)^. The STAI consists of 40 items divided into two subscales: the Trait Anxiety Subscale and the State Anxiety Subscale, each with 20 items. In both subscales, items are rated on a Likert scale ranging from 0 (not at all) to 4 (very much so). Scores can reflect low anxiety (0-30), moderate anxiety (31-44), and high anxiety (≥ 45). Higher scores indicate greater anxiety^(17,18)^. The scale showed good internal consistency, with Cronbach’s alpha ranging from 0.83 to 0.94^(16-18)^.

### Trait Anxiety Subscale (STAI)

The Trait Anxiety Subscale of the State-Trait Anxiety Inventory (STAI) assesses persistent and enduring feelings of anxiety. It was used in one study with pregnant women in the first trimester^(22)^ and with expectant fathers whose partners were in the third trimester^(23)^. It consists of 20 statements, rated on a 4-point Likert scale ranging from 1 (almost never) to 4 (almost always). Items 1, 3, 6, 7, 10, 13, 14, 16, and 19 are reverse-coded. The total score is calculated by summing all items, with higher scores indicating greater anxiety. Internal consistency and reliability were not reported.

### State Anxiety Subscale (STAI)

The psychometric characteristics of the State Anxiety Subscale of the State-Trait Anxiety Inventory (STAI), developed by Spielberger and Gorsuch (1970), were evaluated in a study with pregnant women in the second and third trimesters^(24)^. This subscale assesses the current state of anxiety, which is often associated with a specific event and may be transient or indicative of a change in mental health status, in contrast to trait anxiety, which is an enduring characteristic. The scale demonstrated high internal consistency, with a Cronbach’s alpha of 0.95 when tested in pregnant women^(24)^.

### Pregnancy-Related Anxiety Questionnaire - Revised (PRAQ-R)

The Pregnancy-Related Anxiety Questionnaire (PRAQ) consists of 34 items. It was tested^(25)^ and applied to pregnant women in the first, second, and third trimesters of pregnancy^(18,26-28)^. The PRAQ assesses five domains: 1) fear of childbirth, 2) fear of bearing a child with physical or mental disability, 3) fear of changes and disappointment with the partner, 4) fear of change and concern about maternal mental well-being, and 5) the mother-child relationship. Items are scored from 0 to 4, with higher scores indicating greater anxiety. Internal consistency ranged from 0.89 to 0.90^(18,27)^.

### PRAQ - 17-item short version

The 17-item version of the PRAQ was designed by Wendenburg and applied to pregnant women in the first and second trimesters^(29)^. It assesses the exact five domains as the full 34-item version. Each item is scored from 1 to 7, with a total score ranging from 17 to 119. Cronbach’s alpha ranged from 0.69 to 0.76.

### PRAQ - 10-item short version (PRAQ-R-10)

The 10-item short version of the PRAQ (PRAQ-R-10) was administered to pregnant women in all three trimesters^(30-36)^. This version consolidated the five domains of the original scale into three domains: 1) fear of giving birth, 2) worries about bearing a physically or mentally disabled child, and 3) concern about own appearance. Items are scored on a 5-point Likert scale, with higher values indicating greater levels of pregnancy-related anxiety^(35)^. This version demonstrated good internal consistency, with Cronbach’s alpha ranging from 0.83 to 0.95^(30,34,36)^.

### Hamilton Anxiety Rating Scale (HAM-A)

The Hamilton Anxiety Rating Scale (HAM-A) was applied in the third trimester^(37,38)^ and across all three trimesters^(39)^. It assesses mental agitation, psychological distress, and physical symptoms associated with anxiety. The HAM-A consists of 14 items grouped into the following domains: 1) anxious mood, 2) tension, 3) fears, 4) insomnia, 5) intellectual, 6) depressed mood, 7) somatic (muscular), 8) somatic (sensory), 9) cardiovascular symptoms, 10) respiratory symptoms, 11) gastrointestinal symptoms, 12) genitourinary symptoms, 13) autonomic symptoms, and 14) behavior at interview. Each domain is rated on a 3-point Likert scale from 0 to 2 (never, sometimes, always). The total score ranges from 0 to 56, with higher scores indicating greater anxiety^(37)^. The scale demonstrated good reliability, with a Cronbach’s alpha of 0.85^(39)^.

### Pregnancy-Specific Anxiety Inventory (PSAI)

The Pregnancy-Specific Anxiety Inventory (PSAI) was administered to pregnant women across all three trimesters^(19)^. This instrument evaluates four domains related to pregnancy and childbirth: 1) anxiety about being pregnant, 2) anxiety about childbirth, 3) anxiety about breastfeeding, and 4) anxiety about newborn care. It consists of 40 items scored on a 5-point Likert scale. The instrument showed acceptable internal consistency, with a Cronbach’s alpha of 0.76.

### Self-Rating Anxiety Scale (SAS)

The Self-Rating Anxiety Scale (SAS), developed by Zung in 1971, was applied to pregnant women in the first^(40)^, second^(40,41)^, and third^(40,41)^ trimesters. It consists of 20 self-reported items, each of which is rated on a 4-point Likert scale ranging from 1 (none or a little of the time) to 4 (most or all of the time). Higher scores indicate greater anxiety. The raw score is multiplied by 1.25 to obtain a standard score between 25 and 100, with scores ≥50 indicating the presence of anxiety symptoms^(40)^. Reliability in the included studies ranged from 0.74 to 0.83^(40,41)^.

### Perinatal Anxiety Screening Scale (PASS)

The structure and psychometric properties of the Perinatal Anxiety Screening Scale (PASS) were tested in a sample of 606 pregnant women across all three trimesters^(42)^. The PASS consists of 31 items that evaluate four dimensions of perinatal anxiety symptoms: 1) general worry and specific fears, 2) perfectionism, control, and trauma, 3) social anxiety, and 4) acute anxiety and adjustment. Items are rated on a 4-point Likert scale ranging from 0 (not at all) to 3 (almost always), with a total score ranging from 0 to 93. Higher scores indicate more severe anxiety symptoms^(42)^. All dimensions of the PASS demonstrated good internal consistency, with Cronbach’s alpha ranging from 0.837 to 0.890. The authors concluded that this is a suitable instrument for screening anxiety during pregnancy.

The following instruments were used to assess stress in expectant parents during pregnancy.

### Pregnancy Experience Scale - Brief Version (PES-Brief)

The Pregnancy Experience Scale - Brief Version (PES-Brief) was used to assess stress in the third trimester of pregnancy^(22)^. The original PES includes two subscales: *Uplifts*, which asks women how often ten specific pregnancy-related experiences made them feel happy, positive, or uplifted, and *Hassles*, which asks how often ten experiences made them feel unhappy, negative, or upset. The brief version contains 20 items in total, with 10 items scored only as *Uplifts* and 10 scored only as *Hassles* ^(22)^. Each item is rated on a 4-point Likert scale ranging from 0 (not at all) to 3 (a great deal). The study authors classified internal consistency as high, although Cronbach’s alpha was not reported.

### Pregnancy Stress Rating Scale (PSRS)

The Pregnancy Stress Rating Scale (PSRS) was applied to a group of pregnant women in the second^(43,44)^ and third trimesters^(43)^. It assesses psychological stress during pregnancy across five dimensions: 1) stress from seeking safe passage for mother and child through pregnancy, labor, and delivery, 2) stress related to infant care and changes in family relationships, 3) stress from maternal role identification, 4) stress from social support seeking, and 5) stress from altered physical appearance and body function^(43)^. The PSRS consists of 36 items rated on a Likert scale ranging from 0 (definitely no) to 4 (very severe). It showed good internal consistency, with a Cronbach’s alpha of 0.92^(43)^.

### Antenatal Perceived Stress Inventory (APSI)

A validation study of the Antenatal Perceived Stress Inventory (APSI), developed by Razurel et al. (2014), was conducted in the Turkish population with pregnant women in the third trimester^(45)^. This scale assesses perceived stress during the prenatal period and identifies potential stress factors. It consists of three subscales with 12 items, each rated on a 5-point Likert scale. The total score is calculated by summing all items and dividing by the number of items, with higher scores indicating greater perceived stress. The scale demonstrated acceptable reliability, with a Cronbach’s alpha of 0.70^(45)^.

### Prenatal Psychosocial Profile (PPP)

The Prenatal Psychosocial Profile (PPP) administered to women in the third trimester of pregnancy to assess the extent to which each situation caused stress^(46)^. The scale consists of 11 items and additional elements addressing issues such as the maternal role, maternal satisfaction, and maternal overload, all recognized as potential stressors. The study did not report the psychometric characteristics of this scale^(46)^.

### Perceived Stress Scale (PSS)

The Perceived Stress Scale (PSS) was applied to pregnant women in the first^(22)^, second^(21,24)^, and third trimesters^(24,47)^ to examine its psychometric properties. The PSS measures how often participants felt stressed during the past month. It consists of 10 items rated on a 5-point Likert scale ranging from 1 (never) to 5 (very often), with total scores ranging from 0 to 56. Higher scores indicate greater perceived stress^(21)^. Items 4, 5, 6, 7, 10, and 13 are reverse-scored. The scale showed high internal consistency in the included studies, with Cronbach’s alpha ranging from 0.88 to 0.95^(21,22,24,47)^.

### Prenatal Maternal Stress Scale (PNMS)

The Prenatal Maternal Stress Scale (PNMS) was developed based on a pilot study with pregnant women across all three trimesters^(48)^. This multidimensional instrument measures prenatal maternal stress regardless of trimester, parity, or socioeconomic context, allowing health professionals to intervene in a timely manner. The final version consists of 15 items grouped into four domains: 1) perceived social support, 2) pregnancy-specific concerns (physiological and emotional), 3) intimate partner relations, and 4) financial concerns. The scale demonstrated good internal consistency, with a Cronbach’s alpha of 0.80.

## DISCUSSION

In this scoping review, 16 instruments were identified that were applied to pregnant women and expectant fathers to assess anxiety and stress, either in specific trimesters or across all trimesters of pregnancy (Figures 2 and 3). Ten instruments were identified for assessing anxiety, of which two are shortened versions of the original instrument, and two others are subscales (applied independently) of an instrument also identified. For stress assessment, six instruments were identified.

The most frequently used instruments for assessing anxiety were the PRAQ-R-10, the PRAQ R, and the STAI. Most of the anxiety assessment instruments were developed for or applied across all three trimesters of pregnancy, including the PRAQ-R-10^(30-33,35,36)^, PRAQ-R^(18,24,26-28,34)^, STAI^(16-21)^, HAM-A^(37-39)^, PSAI^(19)^, SAS^(40,41)^, and PASS^(42)^.

Regarding the psychometric characteristics of the studies, it is essential to determine whether the instrument can measure what it is intended to. Reliability (i.e., internal consistency) is assessed using Cronbach’s alpha coefficient, which ranges from 0 to 1: the closer to 1, the higher the reliability among indicators^(49)^. For clinical application, a value of 0.9 or higher is required^(49)^.

The findings indicate that the instrument with the highest internal consistency when applied to pregnant women and expectant fathers to assess anxiety is the PRAQ-R-10, which demonstrated a Cronbach’s alpha of 0.95. However, it has not yet been validated for the Portuguese population. The only instrument validated for use with pregnant Portuguese women was the PASS^(42)^. Since most of the instruments used to assess anxiety in expectant couples are not validated for this group in the Portuguese population, validation of the PRAQ-R-10 is recommended, given its strong internal consistency.

Among the studies on stress in expectant fathers, most focused on the third trimester, and the most frequently used instrument was the PSS^(21,22,24,47)^.

As for the psychometric characteristics of the studies, the instrument with the highest internal consistency for assessing stress in pregnant women and expectant fathers was also the PSS, which showed a Cronbach’s alpha of 0.95. In Portugal, it has already been validated^(50)^, although the sample in the validation study did not meet the inclusion criteria established for this scoping review.

In summary, various countries have developed or applied instruments to assess anxiety and stress in expectant parents during pregnancy; however, their application has mainly focused on the third trimester and on women. Only two studies included men or partners of pregnant women as participants, in which the STAI Trait Anxiety Subscale and the PSS were applied^(21,23)^.

Paternal stress is one of the aspects that should be considered during pregnancy, as this is a critical period for health promotion and complication prevention^(11)^. Increasingly, expectant fathers express concern and anxiety about issues related to pregnancy and maternal and neonatal health, leading them to seek information on these topics through social media and websites^(23)^. It is recommended that they be included in their partners’ prenatal health education, in order to increase their awareness of pregnancy risk factors, childbirth, and the essential importance of their support throughout the entire process, thereby improving maternal and child health^(21)^.

Regarding the study population, most of the studies included women, as this has been the main focus of researchers. Further research on anxiety and stress in expectant fathers is needed to better understand their experiences and needs. The use of assessment instruments will make it possible to plan and implement specific and targeted interventions. Although fathers are allowed to participate in prenatal care visits, not all can do so. Encouraging the inclusion of expectant fathers not only brings them closer to research but also underscores their importance in the pregnancy process.

### Study limitations

Despite the use of a predefined protocol and a methodological strategy for searching the scientific literature, the exclusion of some databases may have resulted in missing relevant studies. Most of the identified assessment instruments were applied in different cultural contexts, particularly in China, the Netherlands, and Turkey; therefore, these tools may not be fully applicable to pregnant women in other parts of the world, especially in the Portuguese population. As mentioned, few studies assessing anxiety and stress in men have been validated for the Portuguese population.

### Contributions to the field

The findings of this review are relevant to the daily clinical practice of health professionals. By applying instruments that assess anxiety and stress, it becomes possible to implement tailored, anticipatory interventions to meet the needs of expectant parents, preventing complications and promoting a positive pregnancy experience. Within this set of interventions, depending on the identified anxiety or stress factor, actions may include coordination with the multidisciplinary team for follow-up and collaboration throughout pregnancy, with the aim of promoting maternal-fetal mental health and well-being, as well as contributing to a positive pregnancy experience.

## CONCLUSIONS

This scoping review identified instruments for assessing anxiety and stress to be applied to expectant parents during pregnancy. In several countries, extensive research on anxiety and stress was observed, with greater focus on pregnant women and in Asia. The most frequently used instruments were the PRAQ and the PSS. However, expectant fathers were a minority among the participants in the studies included in this review, highlighting the limited knowledge about these experiences in men. This gap should be addressed through further research, as fathers are fundamental pillars of maternal well-being, particularly regarding the mother’s emotional stability during pregnancy.

To maximize research on the paternal role, their participation in prenatal visits should be encouraged, where brief screening instruments such as the PSS can be applied. Making instruments available online may facilitate access for health professionals to these men, since many face difficulties attending with their partners or feel reluctant to express their feelings. Incorporating instruments into childbirth and parenting education sessions attended by fathers is also important to normalize the emotional aspects of pregnancy and to reinforce the active role of fathers.

As a transitional period to parenthood, pregnancy is a stage in the lives of expectant parents when they require substantial support and follow-up from nurses. The use of valid and reliable instruments to assess anxiety and stress during this period allows nurses not only to identify these experiences in expectant parents but also to develop a practice based on anticipatory interventions that facilitate the transition to parenthood and promote a positive pregnancy experience.

For future research, assessing the quality of studies may help health professionals select instruments that support clinical assessment. Furthermore, it is essential to develop and validate instruments tailored to the Portuguese population.

## Data Availability

The research data are available within the article.
