# Supplementary material for: Assessment of anxiety and psychological stress among expectant parents during pregnancy: a scoping review
Source: Rev Bras Enferm. 2025 Dec 8;78(6):e20240587. doi: 10.1590/0034-7167-2024-0587 (PMC12707617; doi:10.1590/0034-7167-2024-0587)
Supplement: Translation

# Translation: Avaliação da ansiedade e estresse psicológico dos futuros pais na gravidez: uma revisão de escopo

**DOI:** 10.1590/0034-7167-2024-0587pt

**Authors:** Inês Santos Tinta, Maria Helena de Carvalho Valente Presado, Sandra Margarida Gaiato Risso

**Affiliations:** I Centro de Investigação, Inovação e Desenvolvimento de Enfermagem de Lisboa. Lisboa, Portugal; II Escola Superior de Enfermagem de Lisboa. Lisboa, Portugal

**Keywords:** Ansiedade, Avaliação de Risco, Gravidez, Questionário de Avaliação do Paciente, Estresse Psicológico.

## Abstract

**Objetivos:**

mapear, na literatura científica, instrumentos que permitam avaliar a ansiedade e o estresse dos futuros pais durante a gravidez.

**Métodos:**

revisão de escopo conduzida conforme diretrizes do JBI e PRISMA-ScR, nas bases de dados CINAHL, MEDLINE, Cochrane, Scopus, *ScienceDirect, Web of Science* e Repositórios Científicos de Acesso Aberto de Portugal. Foram incluídos estudos quantitativos sem restrição temporal e de idioma.

**Resultados:**

obtiveram-se 16 instrumentos (10 para avaliação da ansiedade e 6 para avaliação do estresse), predominantemente aplicados às grávidas no terceiro trimestre.

**Conclusões:**

são escassos os estudos com futuros pais homens. Para compreender melhor seus sentimentos e necessidades, é fundamental aplicar instrumentos de avaliação da ansiedade e do estresse, permitindo um cuidado antecipatório. Construir e/ou validar instrumentos com futuros pais (homem e mulher) nos vários trimestres de gravidez permitirá alargar o conhecimento dos profissionais e da população sobre essa problemática.

## INTRODUÇÃO

O processo de gravidez é entendido como um momento de extrema alegria e felicidade, contudo a literatura científica evidencia que, cada vez mais, os futuros pais (homem e mulher) sentem um aumento crescente da ansiedade e do estresse, podendo influenciar negativamente a sua saúde física e mental assim como os desfechos neonatais^(1)^. A gravidez contempla a transição para a parentalidade, podendo gerar imensas preocupações no casal e impactos importantes para sua saúde e bem-estar; ademais, a abordagem desse tema concentra-se, principalmente, nos aspetos funcionais da parentalidade, colocando de lado aqueles relacionados com o bem-estar psicológico dos pais^(2)^. O seu bem-estar é influenciado pela forma como se sentem e como experienciam determinados sentimentos, nomeadamente a ansiedade e o estresse.

A ansiedade, em muitas situações, é utilizada como sinónimo da palavra estresse, contudo essa utilização não está correta, pois esse sentimento é definido como uma resposta emocional em antecipação ao perigo, cuja origem é desconhecida ou não é reconhecida^(3)^. O grau de ansiedade sentido está relacionado com alguns fatores predisponentes, um dos quais é o estado de saúde atual.

O estresse, por sua vez, é um estado de desequilíbrio entre as exigências que ocorrem interna ou externamente num indivíduo e a sua capacidade de enfrentar essas exigências^(3)^. Ele é gerado por experiências negativas e positivas, que requerem ajustes como resposta a diversas mudanças na rotina atual, podendo estar até na etiologia de algumas doenças.

Assim, o estresse pode ser considerado uma pressão externa sobre um indivíduo, sendo a ansiedade a resposta emocional subjetiva a esse estressor.

No que concerne à gravidez, esta pode ser entendida, por si só, como um fator de estresse*¸* uma vez que é caracterizada por alterações neuroendócrinas, cardiovasculares e imunológicas^(4)^. Os futuros pais apresentam maior risco de desenvolver problemas em nível emocional e mental ao longo da gravidez e do primeiro ano após o nascimento do bebé^(5)^.

O rastreio dos sentimentos de ansiedade e de estresse é fundamental, pois a ansiedade materna e paterna durante a gravidez aumenta a probabilidade de problemas de comportamento nas crianças sendo a materna a de maior influência nos resultados^(6)^. Contudo, o estresse paterno nesse período influencia também de forma direta o desenvolvimento infantil^(7)^. No tocante às futuras mães, cerca de 40% das grávidas apresentam sintomas de ansiedade e identificam a possibilidade de distúrbios de estresse durante a gravidez^(4,8)^. O papel do pai também afeta diretamente o estresse da mulher grávida, mostrando a influência mútua de sentimentos^(9)^.

Os futuros pais homens consideram o período de gravidez uma fase difícil, quando são vivenciadas várias emoções como medo e ansiedade, que podem afetar negativamente o relacionamento não só com a esposa, mas também, posteriormente, com seu filho^(10)^. Logo, o estresse paterno é um sentimento a ter também em consideração, devendo ser avaliado na gravidez, pois esta representa um período de ouro para intervir precocemente na prevenção de complicações^(11)^.

Os autores recomendam o desenvolvimento de ações que incluam o rastreio de ansiedade e estresse nos pais homens, de forma a implementar intervenções preventivas e terapêuticas adequadas. Essa indicação justifica-se porque cerca de 25% apresentam sintomas de ansiedade durante a gravidez das suas esposas, com a origem desse sentimento assentando em várias esferas: preocupações específicas com o parto, com a transição para a paternidade e com o bebé, relacionamento com a parceira, atitudes relativas aos profissionais que acompanharão o processo e preocupações financeiras^(11,12)^.

Os pais homens podem proteger contra o desenvolvimento de problemas de saúde mental maternos decorrentes do estresse e ansiedade na gravidez, desde que sua saúde mental seja cuidada mediante a promoção do bem-estar^(13)^. Como visto anteriormente, a presença desses sentimentos nos futuros pais durante o processo de gravidez influencia o comportamento da criança; logo, cuidar da saúde mental do casal por meio da identificação precoce beneficia a família como um todo. Adicionalmente, os homens referem que muitas vezes relutam em expressar os seus sentimentos e a sua necessidade de ajuda por priorizarem as necessidades das suas parceiras e por sentirem-se excluídos pelos serviços de saúde^(13)^. Esse sentimento de exclusão deriva da falta de recursos humanos e de recursos de saúde adaptados aos homens no âmbito abrangente da paternidade e da saúde mental para promoção do seu autocuidado e do seu papel enquanto cuidadores envolvidos^(13)^.

Dessa forma, tal lacuna por parte dos enfermeiros origina uma inquietação pessoal em identificar instrumentos que possam ser utilizados atempadamente para avaliar e detetar ansiedade e estresse nos pais durante a gravidez, possibilitando a adoção de práticas de cuidado que favoreçam uma vivência positiva desse período tão marcante na vida do casal. Para desenvolver a presente temática, o método de investigação mais apropriado é a revisão de escopo, pois permite mapear e sintetizar a evidência científica existente bem como identificar lacunas^(14)^.

Vale pontuar que uma pesquisa preliminar no JBI e nas bases de dados MEDLINE Ultimate^®^ (EBSCOhost) e na Cochrane Database of Systematic Reviews^®^ (EBSCOhost) não identificou revisões sistemáticas ou revisões de escopo atuais ou em desenvolvimento sobre o tema.

## OBJETIVOS

Mapear, na literatura científica, instrumentos para avaliar a ansiedade e o estresse dos futuros pais (homem e mulher) durante a gravidez.

## MÉTODOS

### Desenho e período do estudo

A revisão de escopo foi realizada de acordo com a metodologia do JBI para revisões de escopo, seguindo as diretrizes do *Preferred Reporting Items for Systematic Review and Meta-Analysis extension for Scoping Reviews* (PRISMA-ScR)^(14)^. Nessa metodologia, foi desenhado um protocolo para definir os objetivos, os métodos (incluindo as fontes de evidência, critérios de exclusão, método de extração e de apresentação de dados e análise de evidência) e a estratégia de pesquisa. O protocolo foi registado na Open Science Framework Registries (OSF) a 26 de abril de 2024 e pode ser consultado em https://doi.org/10.17605/OSF.IO/EB3FA. Ele sofreu algumas alterações, nomeadamente no que tange aos critérios de exclusão, tendo sido adicionados dois critérios não mencionados no protocolo inicial (pandemia de COVID-19 e comportamentos aditivos). A pesquisa para a execução da presente revisão de escopo foi efetuada a 30 de abril de 2024 e atualizada a 15 de abril de 2025, não tendo sido identificados mais estudos publicados que cumprissem os critérios de inclusão e que respondessem à questão de pesquisa da presente revisão.

A estratégia de pesquisa teve como objetivo localizar estudos publicados e não publicados, sem limite temporal e sem restrição de idioma. Uma pesquisa inicial limitada à MEDLINE Ultimate^®^ e CINAHL Ultimate^®^ foi realizada para identificar artigos sobre o tema. As palavras do texto contidas nos títulos e resumos dos artigos relevantes assim como as palavras-chave e os termos indexados foram usados para desenvolver, na segunda fase, uma estratégia de pesquisa completa também na Cochrane Database of Systematic Reviews, ScienceDirect^®^, Scopus^®^ e Web of Science^®^. As fontes de estudos não publicados/da literatura cinzenta a serem pesquisadas incluíram os Repositórios Científicos de Acesso Aberto de Portugal, artigos científicos eletrónicos e livros em formato digital. A inclusão de literatura cinzenta reduz a suscetibilidade de viés uma vez que são considerados todos os dados sobre o assunto^(15)^. Foi utilizada a seguinte estratégia de pesquisa: (“*Future Parents*” *OR* “MH *Expectant Mothers*” *OR “MH Expectant Fathers” OR “MH Expectant Parents”) AND ((“Anxiety” OR “MH Anxiety”) OR (“*Estresse*” OR “MH Phychological* Estresse*” OR “MH* Estresse”)) AND (“Pregnancy” OR “Pregnan*” OR “MH Pregnancy”) AND (“Assessment instrument” OR “Assessment tool” OR “Scale*” OR “Questionnaire” OR “Form” OR “MH Risk Assessment”). A expressão de pesquisa incluiu truncaturas, nomeadamente pregnan* e scale*, a fim de abranger o maior número possível de artigos sobre o tema.

Quanto aos critérios de inclusão, foi utilizada a estratégia *Participants, Concept* e *Context* (PCC), sendo que os participantes foram os futuros pais (homem e mulher); os conceitos foram a ansiedade, o estresse e a gravidez; e o contexto considerado foram estudos que incluíssem instrumentos de avaliação.

Todos os artigos científicos sobre o tema foram incluídos, especificamente estudos de abordagem quantitativa, publicados ou não, com desenho de estudos experimentais e quase experimentais, incluindo ensaios clínicos controlados aleatórios e não aleatórios, estudos antes e depois e estudos de séries temporais interrompidos, estudos de caso-controlo, estudos transversais analíticos ou descritivos, estudos de desenvolvimento/validação e revisões sistemáticas que atendam aos critérios de inclusão. Os textos e artigos de opinião também foram considerados para inclusão nesta revisão de escopo assim como artigos em qualquer idioma, sem limitação temporal.

Foram excluídos os métodos mistos, cartas, resumos em anais de eventos, artigos incompletos ou estudos em andamento. Também foram excluídos estudos cujo conceito fosse depressão, depressão pós parto, estresse patológico ou ansiedade patológica, gravidezes com patologia induzida ou associada, gravidezes resultantes de técnicas de procriação medicamente assistida ou fetos com patologia diagnosticada *in utero* e estudos que abordassem a pandemia de COVID-19 e comportamentos aditivos.

Na terceira fase, após a pesquisa, todas as citações identificadas foram reunidas e carregadas no programa Rayyan^®^; e os duplicados, removidos. Dois revisores independentes avaliaram os títulos e resumos em relação aos critérios de inclusão da revisão. As fontes potencialmente relevantes foram recuperadas na íntegra. Novamente dois revisores independentes avaliaram detalhadamente o texto completo das citações selecionadas de acordo com os critérios de inclusão. As divergências foram resolvidas sem necessidade de recorrer a um revisor adicional.

## RESULTADOS

Foram identificados 1.591 estudos. Após transferência para o aplicativo Rayyan^*®*^, foram removidos 49 duplicados, e 4 artigos foram automaticamente excluídos pelo critério de inelegibilidade, resultando em 1.538 artigos para análise. Foram ainda identificados mais dois artigos na literatura cinzenta, contudo um foi excluído na primeira fase de análise. Após a aplicação dos critérios de inclusão e de exclusão, foram excluídos 1.484 e incluídos 55 artigos para análise integral, dos quais 22 foram excluídos. Portanto, a presente revisão de escopo contempla 33 artigos. Os resultados da pesquisa e do processo de inclusão e de exclusão estão apresentados na Figura 1 em forma de diagrama de fluxo com base no *Preferred Reporting Items for Systematic Reviews and Meta Analyses Extension for Scoping Review* (PRISMA-ScR).

**Figura 1:** Diagrama de fluxo com base no *Preferred Reporting Items for Systematic Reviews and Meta-Analyses Extension for Scoping Review*.

Os dados extraídos dos artigos incluídos foram organizados numa tabela de extração de dados desenvolvida e adaptada segundo as orientações do JBI. É apresentada da seguinte forma: autores/ano/país, objetivos, participantes, métodos (desenho do estudo), instrumentos para avaliação da ansiedade, instrumentos para avaliação do estresse e características psicométricas.

Os dados extraídos dos estudos foram organizados de acordo com o instrumento de avaliação de ansiedade e estresse identificado e com o trimestre de gravidez em que ele foi utilizado (primeiro, segundo ou terceiro trimestre). Os resultados encontram-se apresentados em gráficos, figuras e tabelas.

### Data e local de publicação

Os estudos incluídos (n = 33) foram publicados entre 2004 e 2024. O número de publicações aumentou desde 2019 (n = 5) e atingiu o pico em 2022 (n = 7), evidenciando o crescimento da produção científica sobre a temática e uma atualização contínua da evidência.

Os estudos que incluem instrumentos de avaliação da ansiedade e do estresse foram desenvolvidos em vários países (Figura 2), nomeadamente China (n = 6; 19%), Holanda (n = 5; 16%), Turquia (n = 4; 12%), Estados Unidos (n = 3; 9%), Canadá (n = 2; 6%), Índia (n = 2; 6%), Indonésia (n = 2; 6%), Irão (n = 2; 6%), Austrália (n = 1; 3%), Brasil (n = 1; 3%), Escócia (n = 1; 3%), Espanha (n = 1; 3%), Finlândia (n = 1; 3%), Portugal (n = 1; 3%) e Iraque (n = 1; 3%). Destaca-se a Ásia (representada por China, Turquia, Índia e Indonésia) como o continente com o maior interesse na investigação da ansiedade e do estresse.

**Figura 2:** Distribuição da percentagem de estudos por país

### Tipos de estudos

Os estudos analisados contêm uma abordagem quantitativa. Na sua maioria, são longitudinais (n = 8), seguidos de correlacionais (n = 7), transversais (n = 6), quase experimentais (n = 7), experimentais (n = 2), de validação (n = 4), exploratório (n = 1), piloto (n = 1) e de replicação (n = 1). Assim, os desenhos de estudo mais comuns foram os longitudinais prospetivos.

### Objetivos dos estudos

O objetivo principal dos trabalhos é determinar a prevalência de ansiedade e de estresse em mulheres grávidas durante os três trimestres de gravidez. Algumas pesquisas comparam, nas mulheres grávidas, os níveis de ansiedade geral e específica na gravidez e primeiro mês de vida da criança com a qualidade de vida, satisfação com a vida e prováveis fatores associados, como fatores culturais, relação com o pai e qualidade do sono. Examinam ainda os fatores preditores de estresse, relacionando o estresse nos futuros pais com o aconselhamento durante a gravidez e associando a ansiedade com a busca de informação sobre a gravidez. Alguns estudos desenvolvem, traduzem, validam e/ou adaptam os instrumentos para a população do país de origem, nomeadamente Holanda, Austrália, Turquia, Índia, Canadá, China e Estados Unidos.

### Participantes, conceito e contexto

No tocante aos participantes, 31 estudos incluem mulheres grávidas em sua pesquisa, sendo que apenas 2 têm como participantes os futuros pais homens. Quanto à dimensão da amostra, varia entre 19 e 4.541, havendo um estudo com 10.002 participantes.

Entre os 33 artigos incluídos, os autores aplicam 16 instrumentos (descritos detalhadamente de seguida) para avaliação da ansiedade e do estresse nos futuros pais durante os três trimestres de gravidez. A Figura 3 mostra a distribuição da aplicação dos instrumentos para avaliação da ansiedade dos futuros pais no primeiro, segundo e terceiro trimestres de gravidez, enquanto a Tabela 1 constitui uma tabela-resumo comparando os mesmos instrumentos em termos de aplicabilidade, consistência interna (alfa de Cronbach) e trimestres de aplicação. A Figura 4 mostra a distribuição da aplicação dos instrumentos para avaliação do estresse dos futuros pais no primeiro, segundo e terceiro trimestres de gravidez, ao passo que a Tabela 2 mostra-se como uma tabela-resumo comparando os mesmos instrumentos em termos de aplicabilidade, consistência interna (alfa de Cronbach) e trimestres de aplicação.

**Tabela 1:** Tabela-resumo de comparação dos instrumentos para avaliação da ansiedade em termos de aplicabilidade, consistência interna (alfa de Cronbach) e trimestres de aplicação

Instrumento	Aplicabilidade		Consistência interna (alfa de Cronbach)	Trimestre de aplicação		
	Mulheres	Homens		1º	2º	3º
**Inventário de Ansiedade Traço-Estado (IDATE)**	x	x	0,83-0,94	x	x	x
**Subescala de Ansiedade-Traço (IDATE)**	x	x	Não descrito	x		x
**Subescala de Ansiedade-Estado (IDATE)**	x		0,95		x	x
**Escala de Avaliação de Ansiedade Relacionada à Gravidez - Revista (PRAQ-R)**	x		0,89-0,90	x	x	x
**PRAQ - versão resumida 17 itens**	x		0,69-0,76	x	x	
**PRAQ - versão resumida 10 itens**	x		0,83-0,95	x	x	x
**Escala de Avaliação da Ansiedade de Hamilton (HAM-A)**	x		0,85	x	x	x
**Inventário de Ansiedade Específica na Gravidez (PSAI)**	x		0,76	x	x	x
**Escala de Autoavaliação da Ansiedade (SAS)**	x		0,74-0,83	x	x	x
**Escala de Avaliação da Ansiedade Perinatal (ERAP)**	x		0,84-0,89	x	x	x

**Tabela 2:** Tabela-resumo de comparação dos instrumentos para avaliação do estresse em termos de aplicabilidade, consistência interna (alfa de Cronbach) e trimestres de aplicação

Instrumento	Aplicabilidade		Consistência interna (alfa de Cronbach)	Trimestre de aplicação		
	Mulheres	Homens		1º	2º	3º
**Escala de Experiência de Gravidez, versão resumida (PES)**	x		Não especificado			x
**Escala de Avaliação de Estresse na Gravidez (PSRS)**	x		0,92		x	x
**Escala de Estresse Pré-Natal (APSI)**	x		0,70			x
**Escala de Perfil Psicossocial Pré-Natal (PPP)**	x		Não especificado			x
**Escala de Estresse Percebido (PSS)**	x		0,88-0,95	x	x	x
**Escala de Avaliação do Estresse Materno Pré-Natal (PNMS)**	x		0,80	x	x	x

**Figura 3:** Distribuição da aplicação dos instrumentos para avaliação da ansiedade dos futuros pais no primeiro, segundo e terceiro trimestres de gravidez

**Figura 4:** Distribuição da aplicação dos instrumentos para avaliação do estresse dos futuros pais (homem e mulher) no primeiro, segundo e terceiro trimestres de gravidez

### Inventário de Ansiedade Traço-Estado (IDATE)

O Inventário de Ansiedade Traço-Estado (IDATE) foi aplicado a mulheres grávidas no primeiro^(16-20)^, segundo^(17-21)^ e terceiro trimestres^(17-20)^. O IDATE é composto por 40 itens, divididos em duas subescalas: a subescala de ansiedade-traço e a subescala de ansiedade-estado, cada uma composta por 20 questões. Em ambas as subescalas, cada questão é pontuada mediante uma escala Likert de 0 (nada) a 4 (muito), podendo existir baixos níveis de ansiedade (pontuação de 0 a 30), ansiedade moderada (entre 31 e 44) e ansiedade elevada (pontuações acima de 45). Pontuações mais altas representam maior nível de ansiedade^(17,18)^. A escala apresenta boa consistência interna, com alfa de Cronbach a variar entre 0,83 e 0,94^(16-18)^.

### Subescala de Ansiedade-Traço (IDATE)

A Subescala de Ansiedade-Traço do Inventário de Ansiedade Traço-Estado (IDATE) avalia os sentimentos de ansiedade duradoura e persistente, tendo sido utilizada num estudo com mulheres grávidas no primeiro trimestre de gravidez^(22)^ e em futuros pais homens cujas mulheres se encontravam no terceiro trimestre^(23)^. É composta por 20 afirmações, avaliadas em uma escala Likert de 1 (quase nunca) a 4 (quase sempre), na qual os itens 1, 3, 6, 7, 10, 13, 14, 16 e 19 são codificados inversamente. A pontuação total é obtida pela soma de todos os itens, em que valores mais altos indicam maior ansiedade. Não foi descrito o nível de consistência interna e de fiabilidade.

### Subescala de Ansiedade-Estado (IDATE)

As características psicométricas da Subescala de Ansiedade-Estado do Inventário de Ansiedade Traço-Estado (IDATE), de Speilberger e Gorsuch (1970), foram avaliadas num estudo com mulheres grávidas no segundo e terceiro trimestres de gestação^(24)^. Essa subescala visa avaliar o estado atual de ansiedade de um indivíduo, que muitas vezes está associado a um evento específico, podendo ser transitório ou indicativo de mudança no estado de saúde mental, em oposição ao estado característico de ansiedade, denominado “traço” de ansiedade. A escala tem boa consistência interna, apresentando um alfa de Cronbach de 0,95 quando testada em grávidas^(24)^.

### Escala de Avaliação de Ansiedade Relacionada à Gravidez - Revista (PRAQ-R)

A Escala de Avaliação da Ansiedade Relacionada à Gravidez (PRAQ) tem 34 itens. Foi testada^(25)^ e aplicada a mulheres grávidas no primeiro, segundo e terceiro trimestres de gravidez^(18,26-28)^. A PRAQ avalia cinco domínios: 1) medo do parto, 2) medo de gerar um filho com deficiência física ou mental, 3) medo de mudanças e de desilusão com o parceiro, 4) medo da mudança e preocupação com o bem-estar mental e 5) relação mãe-filho. A pontuação varia entre 0 e 4: quanto mais elevada, maior o nível de ansiedade. A consistência interna foi de 0,89-0,90^(18,27)^.

### PRAQ - versão resumida 17 itens

A versão de 17 itens da PRAQ foi desenhada por Wendenburg e aplicada a mulheres grávidas no primeiro e segundo trimestres de gravidez^(29)^. Avalia os mesmos cinco domínios da versão completa de 34 itens. Cada item é pontuado de 1 a 7, e a soma total vai de 17 a 119. O alfa de Cronbach variou de 0,69 a 0,76.

### PRAQ - versão resumida 10 itens

A versão da PRAQ resumida a 10 itens (PRAQ-R-10) foi aplicada a mulheres grávidas nos três trimestres de gravidez^(30-36)^. Os três fatores avaliados nessa versão são: 1) o medo do parto, 2) o medo de ter um filho com deficiência física ou mental e 3) a preocupação com a aparência. É composta por 10 itens, classificados de 1 a 5: valores maiores indicam níveis mais elevados de ansiedade relacionada à gravidez^(35)^. Essa versão apresentou boa consistência interna, com alfa de Cronbach variando entre 0,83 e 0,95^(30,34,36)^.

### Escala de Avaliação da Ansiedade de Hamilton (HAM-A)

A Escala de Avaliação da Ansiedade de Hamilton foi aplicada tanto no terceiro trimestre^(37,38)^ quanto nos três trimestres^(39)^. Ela busca avaliar a agitação mental, o sofrimento psicológico e as queixas físicas relacionadas com a ansiedade. É composta por 14 itens distribuídos em 14 domínios: 1) humor ansioso, 2) tensão, 3) medos, 4) insónia, 5) intelectual, 6) humor depressivo, 7) somático (muscular), 8) somático (sensorial), 9) sintomas cardiovasculares, 10) sintomas gastrointestinais, 11) sintomas respiratórios, 12) sintomas genitourinários, 13) sintomas autonómicos e 14) comportamento durante a entrevista. Esses domínios são classificados em uma escala de Likert de 0 a 2 (sempre, às vezes e nunca). A pontuação total é obtida pela soma dos 14 itens, variando entre 0 e 56: pontuações mais altas indicam níveis mais elevados de ansiedade^(37)^. A escala apresentou um bom nível de confiabilidade, com alfa de Cronbach igual a 0,85^(39)^.

### Inventário de Ansiedade Específica na Gravidez (PSAI)

O Inventário de Ansiedade Específica na Gravidez foi aplicado a um grupo de mulheres nos três trimestres de gravidez^(19)^. Esse instrumento avalia quatro domínios da gravidez e do parto: 1) ansiedade por estar grávida, 2) ansiedade do parto, 3) ansiedade relacionada com a amamentação e 4) ansiedade com os cuidados ao recém-nascido. É composta por 40 itens, pontuados em uma escala de Likert de 5 pontos. Apresentou um alfa de Cronbach igual a 0,76.

### Escala de Autoavaliação da Ansiedade (SAS)

A Escala de Autoavaliação de Ansiedade (SAS), desenvolvida por Zung em 1971, foi aplicada a mulheres grávidas no primeiro^(40)^, segundo^(40,41)^ e terceiro^(40,41)^ trimestres de gravidez. É composta por 20 itens, e cada item é respondido dentro de uma escala de 1 (nenhuma ou poucas vezes) a 4 (a maior parte ou todas as vezes). Uma pontuação mais alta indica um maior nível de ansiedade. A pontuação total é multiplicada por 1,25 para chegar a valores padronizados entre 25 e 100. Uma pontuação-padrão superior ou igual a 50 indica a presença de sintomas de ansiedade^(40)^. O nível de confiabilidade nos estudos incluídos variou de 0,74 a 0,83^(40,41)^.

### Escala de Avaliação da Ansiedade Perinatal (ERAP)

A estrutura e as características psicométricas da Escala de Avaliação da Ansiedade Perinatal (ERAP) foram testadas num grupo de 606 mulheres grávidas durante os três trimestres de gravidez^(42)^. Essa escala é composta por 31 itens que avaliam quatro dimensões de sintomatologia ansiosa no período perinatal: 1) preocupação excessiva e medos específicos, 2) perfeccionismo, controlo e trauma, 3) ansiedade social, e 4) ansiedade aguda e ajustamento. Os itens são avaliados em uma escala Likert que varia entre 0 (nunca) e 3 (quase sempre), em que a pontuação total vai de 0 a 93. Quanto mais elevada a pontuação, mais grave é a sintomatologia^(42)^. Todas as dimensões da ERAP demonstraram uma boa consistência interna, com alfa de Cronbach entre 0,837 e 0,890. Os mesmos autores concluem que este é um bom instrumento para rastreio da ansiedade na gravidez.

De seguida, serão apresentados os instrumentos para avaliação do estresse dos futuros pais (homem e mulher) durante a gravidez.

### Escala de Experiência de Gravidez, versão resumida (PES)

A versão resumida da Escala de Experiência de Gravidez foi aplicada para avaliar o estresse no terceiro trimestre de gravidez^(22)^. A PES original contém duas subescalas: a *Uplifts,* que questiona as mulheres sobre a frequência com que dez experiências específicas as fazem sentir felizes e positivas; e a subescala *Hassles,* que questiona sobre a frequência com que dez experiências as fazem sentir negativas e infelizes. A versão resumida é composta apenas por dez itens que contemplam as principais experiências positivas e negativas^(22)^. Para cada item, a opção de resposta varia de 1 (não, absolutamente) a 4 (totalmente). No que concerne à consistência interna, os autores do estudo classificam-na como elevada, não especificando o alfa de Cronbach.

### Escala de Avaliação de Estresse na Gravidez (PSRS)

A Escala de Avaliação de Estresse na Gravidez (PSRS) foi aplicada a um grupo de mulheres grávidas no decorrer do segundo^(43,44)^ e terceiro trimestres de gravidez^(43)^. Avalia o estresse psicológico na gravidez em cinco dimensões: 1) estresse por procurar uma passagem segura para a mãe e feto durante a gravidez, trabalho de parto e parto, 2) estresse relacionado aos cuidados com o bebê e mudança nas relações familiares, 3) estresse relacionado à identificação do papel materno, 4) estresse resultante da busca de apoio social e 5) estresse decorrente da alteração da aparência física e na funcionalidade corporal^(43)^. É composta por 36 itens avaliados numa escala Likert que varia de 0 (definitivamente não) a 4 (muito severo). Apresentou boa consistência interna, com alfa de Cronbach igual a 0,92^(43)^.

### Escala de Estresse Pré-Natal (APSI)

Foi efetuado um estudo de validação da Escala de Estresse Pré-Natal, de Razurel et al. (2014), para a população turca com mulheres grávidas no terceiro trimestre de gravidez^(45)^. Essa escala, segundo os autores do estudo de validação, avalia o estresse percebido no período pré-natal, determinando ainda os fatores de estresse. É composta por 3 subescalas, com 12 itens, pontuadas com uso de uma escala Likert de 5 pontos. A pontuação total é obtida pela soma das pontuações, dividida pelo número de itens: quanto mais elevada a pontuação, maior o nível de estresse percebido. A escala apresentou uma elevada confiabilidade, com alfa de Cronbach de 0,70^(45)^.

### Escala de Perfil Psicossocial Pré-Natal (PPP)

A Escala de Perfil Psicossocial Pré-Natal foi aplicada a mulheres no terceiro trimestre de gravidez para avaliar o quanto cada situação apresentada causava estresse^(46)^. A escala é composta por 11 itens e um conjunto de elementos que abordam questões como o papel da maternidade e a satisfação e a sobrecarga materna, reconhecidas como potenciais geradoras de estresse. O estudo não especifica as características psicométricas da presente escala^(46)^.

### Escala de Estresse Percebido (PSS)

A Escala de Estresse Percebido (PSS) foi aplicada a mulheres grávidas no primeiro^(22)^, segundo^(21,24)^ e terceiro trimestres de gravidez^(24,47)^ para avaliar suas características psicométricas. Essa escala avalia com que frequência o participante se sentiu estressado no último mês. É composta por dez itens avaliados em uma escala Likert de 1 (nunca) a 5 (muitas vezes), com pontuação total variando entre 0 e 56: pontuações elevadas indicam maior nível de estresse^(21)^. Os itens 4, 5, 6, 7, 9, 10 e 13 são pontuados inversamente. A escala teve um nível elevado de consistência interna, com alfa de Cronbach a variar entre 0,88 e 0,95 nos estudos incluídos^(21,22,24,47)^.

### Escala de Avaliação do Estresse Materno Pré-Natal (PNMS)

A Escala de Avaliação do Estresse Materno Pré-Natal (PNMS) foi desenvolvida com base num teste-piloto efetuado com mulheres grávidas nos três trimestres de gravidez^(48)^. Essa escala tem uma abordagem multidimensional que permite mensurar o estresse pré-natal em grávidas independentemente do trimestre, paridade e contexto socioeconómico, para que os profissionais de saúde consigam intervir oportunamente. É composta por 15 itens, que assentam em 4 categorias: 1) apoio social percebido, 2) preocupações específicas da gravidez (fisiológico e emocional), 3) relações íntimas com os parceiros e 4) preocupações financeiras. Segundo os autores do estudo supracitado, a escala teve uma elevada consistência interna, com um alfa de Cronbach de 0,80.

## DISCUSSÃO

Na presente revisão de escopo, foram identificados 16 instrumentos aplicados a mulheres grávidas e a futuros pais homens para avaliar a ansiedade e o estresse, envolvendo trimestres específicos ou todos os trimestres de gravidez (Figuras 2 e 3). Foram identificados dez instrumentos para avaliar a ansiedade, dos quais dois são versões resumidas do instrumento original, e outros dois são subescalas (aplicadas isoladamente) de um instrumento também identificado. Já para avaliar o estresse, foram identificados seis instrumentos.

Os instrumentos mais utilizados para avaliação da ansiedade foram a PRAQ-R-10, a PRAQ R e a STAI/IDATE. A maioria dos instrumentos para avaliar a ansiedade foram desenvolvidos ou aplicados durante os três trimestres de gravidez, como é o caso da PRAQ-R-10^(30-33,35,36)^, PRAQ R^(18,24,26-28,34)^, STAI/IDATE^(16-21)^, HAM-A^(37-39)^, PSAI^(19)^, SAS^(40,41)^ e ERAP^(42)^.

No que concerne às características psicométricas dos estudos, é importante saber se o instrumento consegue inferir ou medir aquilo a que se propõe. Por isso, é preciso avaliar a sua confiabilidade (i.e., a sua consistência interna) por meio do coeficiente alfa de Cronbach, que varia de 0 a 1: quanto mais próximo de 1, maior a confiabilidade entre os indicadores^(49)^. Para a aplicação clínica, é necessário um valor igual ou superior a 0,9^(49)^.

Conclui-se que o instrumento com maior consistência interna ao ser aplicado em grávidas e futuros pais homens para a avaliação da ansiedade é a PRAQ-10, já que apresentou alfa de Cronbach de 0,95. Contudo, ainda não existe validação para a população portuguesa. O único validado para a população portuguesa grávida encontrado foi a ERAP^(42)^. Como a maioria dos instrumentos utilizados para avaliar a ansiedade em casais grávidos não são validados para esse grupo da população portuguesa, sugere-se a validação da PRAQ-10 uma vez que apresenta boa consistência interna.

Entre os estudos sobre estresse em futuros pais, a maioria focalizou o terceiro trimestre, e o instrumento mais utilizado foi a PSS^(21,22,24,47)^.

Relativamente às características psicométricas dos estudos, conclui-se que o instrumento com maior consistência interna para avaliar o estresse em grávidas e futuros pais homens é também a PSS, visto que apresenta um alfa de Cronbach de 0,95. Em Portugal, ele já se encontra validado^(50)^, porém a amostra do estudo de validação não cumpre os critérios de inclusão definidos para a presente revisão de escopo.

Em suma, salienta-se que, em vários países, existem diversos instrumentos para avaliar a ansiedade e o estresse dos futuros pais durante a gravidez, entretanto a sua aplicação concentrou-se no terceiro trimestre e na população feminina. Apenas dois estudos incluíram homens ou cônjuges de mulheres grávidas como participantes, aos quais foram aplicadas a Subescala de Ansiedade-Traço do IDATE e a PSS^(21,23)^.

O estresse paterno é um dos sentimentos que deve ser considerado durante a gravidez, já que é um período de excelência para a promoção da saúde e prevenção de complicações^(11)^. Cada vez mais, os futuros pais homens mostram-se preocupados e ansiosos com as questões relacionadas à gravidez e saúde materna e neonatal, levando-os a procurar informações sobre esses temas nas redes sociais e em websites^(23)^. É recomendado que eles sejam integrados no processo de educação para a saúde na gravidez das suas parceiras, por forma a aumentar a sua consciencialização sobre fatores de risco na gravidez, sobre o parto e sobre a importância fundamental do seu apoio durante todo esse processo, melhorando a saúde das mães e dos filhos^(21)^.

No tocante à população, a maioria dos estudos teve como participantes mulheres, pois este tem sido o foco dos investigadores. São necessários mais trabalhos sobre o tema da ansiedade e estresse nos futuros pais homens, para que seja possível compreender melhor as suas experiências e necessidades. A aplicação de instrumentos de avaliação permitirá planear e implementar intervenções específicas e direcionadas. Apesar da possibilidade de os pais homens participarem das consultas de vigilância da gravidez, nem todos o conseguem aproveitar. Incentivar a inclusão de futuros pais homens permite não só aproximá-los da investigação, mas também evidenciar a sua importância no processo de gravidez.

### Limitações do estudo

Apesar da adoção de um protocolo prévio e de uma estratégia metodológica para a pesquisa na literatura científica, a não inclusão de todas as bases de dados existentes pode ter resultado na ausência de alguns estudos relevantes. A maioria dos instrumentos de avaliação identificados foram utilizados em diferentes contextos culturais, nomeadamente na China, Holanda e Turquia; logo, essas ferramentas podem não se aplicar às mulheres grávidas noutras partes do mundo, nomeadamente à população portuguesa. Conforme mencionado, são escassos os estudos que avaliaram a ansiedade e o estresse em homens e que, ao mesmo tempo, tenham sido validados para a população portuguesa.

### Contribuições para a área

Os resultados encontrados na presente revisão são importantes para a prática clínica diária dos profissionais de saúde. Isso porque, por meio da aplicação de instrumentos que avaliem a presença de ansiedade e estresse, é possível implementar intervenções personalizadas e antecipatórias das necessidades dos futuros pais, prevenindo complicações e promovendo uma experiência de gravidez positiva. No leque de intervenções, de acordo com a identificação do fator de ansiedade/estresse, inclui-se a articulação com a equipa multidisciplinar para acompanhamento e colaboração durante o processo de gravidez, a fim de promover a saúde mental e bem-estar materno fetal, bem como contribuir para uma experiência positiva da gravidez.

## CONCLUSÕES

A presente revisão de escopo permitiu identificar os instrumentos de avaliação da ansiedade e do estresse para aplicação aos futuros pais (homem e mulher) durante a gravidez. Verificou-se extensa pesquisa no âmbito da ansiedade e do estresse em vários países, com maior incidência nas mulheres grávidas e no continente asiático. Os instrumentos mais utilizados foram a PRAQ e a PSS. Todavia, o futuro pai (homem) apresenta-se como uma minoria na amostra de participantes dos estudos incluídos nesta revisão, revelando a escassez de conhecimento sobre a presença desses sentimentos na figura masculina. Essa lacuna deverá ser colmatada com a realização de mais investigações, uma vez que o pai se apresenta como um pilar fundamental para o bem-estar materno, nomeadamente para a estabilidade emocional da mãe ao longo da gravidez.

Para maximizar a investigação sobre a figura paterna, deve ser incentivada a sua participação nas consultas pré-natais, nas quais sejam aplicados instrumentos de rastreio de resposta curta, como a PSS. A disponibilização on-line dos instrumentos pode facilitar o acesso dos profissionais a esses homens, uma vez que a maioria tem dificuldade em acompanhar a mulher ou sente receio em expressar os seus sentimentos. A incorporação de instrumentos durante sessões de preparação para o parto e parentalidade nas quais o pai esteja presente é também importante para normalizar a abordagem emocional da gravidez, reforçando o papel ativo da figura paterna.

Enquanto período de transição para a parentalidade, a gravidez constitui uma das fases da vida dos futuros pais em que eles necessitam de bastante apoio e acompanhamento por parte dos enfermeiros. Assim, utilizar instrumentos válidos e confiáveis de avaliação da ansiedade e do estresse pelos enfermeiros durante esse período permite que esses profissionais não só identifiquem tais sentimentos nos futuros pais, como também desenvolvam uma prática assente em intervenções antecipatórias que facilitem a transição para a parentalidade e promovam uma experiência de gravidez positiva.

Para pesquisas posteriores, pode ser benéfica a avaliação da qualidade dos estudos por forma a ajudar esses profissionais na seleção de um instrumento para apoiar o diagnóstico. Adicionalmente, mostra-se fundamental construir e/ou validar instrumentos adequados e adaptados à população portuguesa.

## Data Availability

Os dados da pesquisa estão disponíveis no corpo do artigo.
